# Chloro({2-[mesityl(quinolin-8-yl-κN)boryl]-3,5-dimethyl-phenyl}methyl-κC)palladium(II) as a Catalyst for Heck Reactions

**DOI:** 10.3390/molecules200712979

**Published:** 2015-07-17

**Authors:** Sem Raj Tamang, James D. Hoefelmeyer

**Affiliations:** Department of Chemistry, University of South Dakota, 414 E. Clark St., Vermillion, SD 57069, USA; E-Mail: sem.tamang@coyotes.usd.edu

**Keywords:** Heck reaction, catalysis, organometallic, palladium, frustrated Lewis pair, non-classical ligand

## Abstract

We recently reported an air and moisture stable 16-electron borapalladacycle formed upon combination of 8-quinolyldimesitylborane with bis(benzonitrile)dichloropalladium(II). The complex features a tucked mesityl group formed upon metalation of an *ortho*-methyl group on a mesityl; however it is unusually stable due to contribution of the boron p_z_ orbital in delocalizing the carbanion that gives rise to an η^4^-boratabutadiene fragment coordinated to Pd(II), as evidenced from crystallographic data. This complex was observed to be a highly active catalyst for the Heck reaction. Data of the catalyst activity are presented alongside data found in the literature, and initial comparison reveals that the borapalladacycle is quite active. The observed catalysis suggests the borapalladacycle readily undergoes reductive elimination; however the Pd(0) complex has not yet been isolated. Nevertheless, the ambiphilic ligand 8-quinolyldimesitylborane may be able to support palladium in different redox states.

## 1. Introduction

**Scheme 1:**
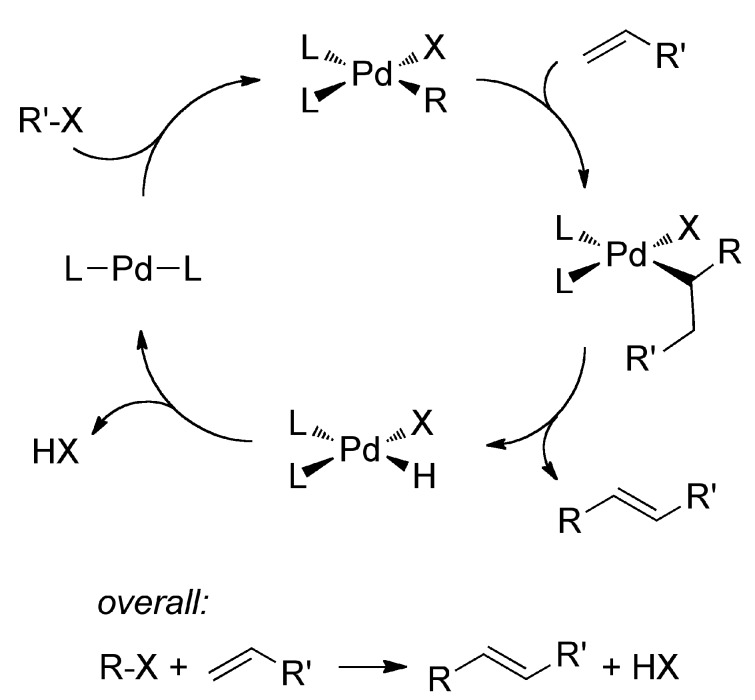
Mechanism of the palladium-catalyzed Heck reaction.

Palladium catalyzed Heck reactions have become an indispensable tool for the synthesis of organic molecules [1,2,3]. The Heck reaction is a C-C coupling, most commonly with an aryl halide and an alkene as reactants (Scheme 1). In the palladium catalyzed reaction, the aryl halide undergoes oxidative addition to a coordinatively unsaturated Pd(0) fragment, followed by 1,2-insertion of the alkene into the Pd-C bond and β-hydride elimination to form the coupled η^2^-alkene, and finally reductive elimination of H-X to regenerate the active Pd(0) fragment. A stoichiometric amount of base is required to neutralize the H-X product. The first palladium catalysts were simple Pd(II) salts, PdCl_2_ or Pd(OAc)_2_. In the course of the reaction, Pd(II) must be reduced to Pd(0) in order to achieve facile oxidative addition of the aryl halide. Ligands could be introduced to stabilize Pd(0) as molecular species and avoid precipitation of the bulk metal. Phosphines are known to stabilize Pd(0), and complexes can be isolated such as Pd(PPh_3_)_4_ [4], Pd(P(*t*Bu)_3_)_3_ [5], and Pd(PCy_3_)_3_ [6]. As a result, phosphines have been a popular choice as a ligand; though, numerous examples of Pd-catalyzed Heck coupling with non-phosphine ligands are known [7,8,9,10,11,12,13,14]. In an effort to arrive at nucleophilic, coordinatively unsaturated Pd(0) centers, the preparation of pre-formed L_2_Pd catalysts with bulky, basic ligands such as phosphines and N-heterocyclic carbenes have been an active area of work [1,2,3]. The steric and electronic properties of these ligands can be varied upon changing the substituents, and the shape of the ligands can impart stereoselectivity or regioselectivity in the catalysis. Also of interest, bent, low-coordinate, nucleophilic metals are more active towards oxidative addition [15], and this may become a new design strategy for future catalysts.

**Scheme 2:**
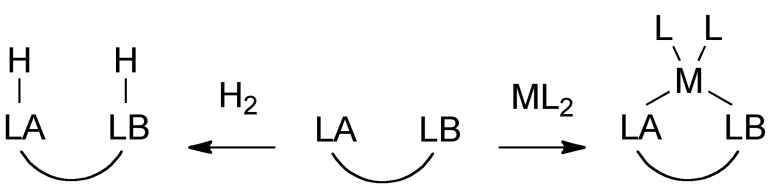
Selected examples of reactions that relieve frustration in FLP molecules via heterolytic bond dissociation or complexation with an ambiphilic guest.

There has been a recent surge of interest in frustrated Lewis pairs (FLPs) that feature Lewis acid-Lewis base combinations that are unable to approach closely to form a strong dative bond to one another [16,17,18]. The frustrated state can be relieved in the presence of appropriate small molecules that undergo heterolytic bond dissociation or ambiphilic molecular fragments (Scheme 2). For example, classic papers from the Stephan lab show that molecular hydrogen undergoes heterolytic cleavage with frustrated phosphine-borane combinations [19,20], and contributed a dramatic increase in interest in the FLP field. Since those findings, it has been shown that several small molecules undergo heterolytic bond dissociation (or severe polarization) in the presence of an FLP, such as CO_2_ [21,22,23,24,25,26], HX [27], X_2_ [28,29], M-X [30], RS-SR [31]. The stabilization of ambiphilic molecules or fragments with a FLP is less explored. The most widely studied examples utilize a unimolecular preorganized FLP as an L-Z ligand to stabilize a transition metal in low oxidation state [32,33]. In this configuration, the Lewis base is formally a neutral two-electron donor (L) to the metal and the Lewis acid is a two-electron acceptor (Z). There is only appreciable contribution of electron density from the metal to the Lewis acid if the metal is in low oxidation state.

**Figure 1:**
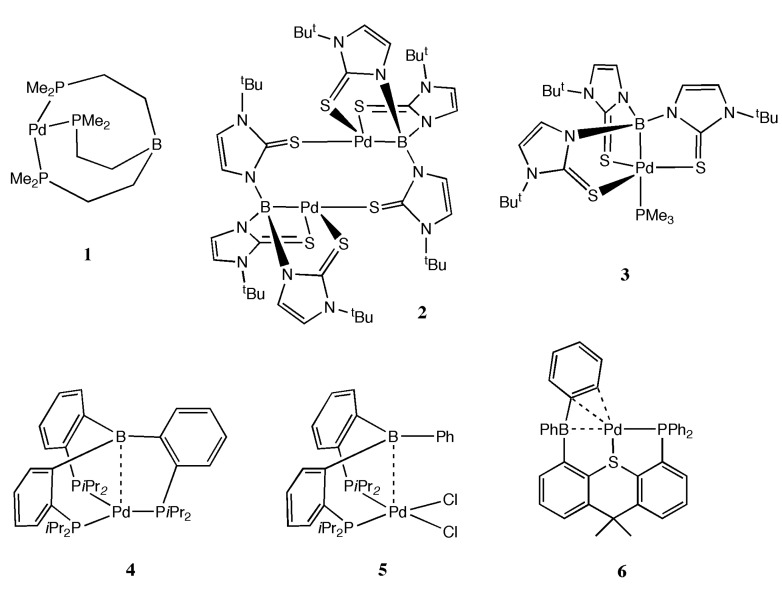
Known L-Pd-Z complexes as reported in the literature.

The use of L-Z ligands to stabilize palladium may be an interesting avenue of investigation, especially considering the potential utility of such complexes for catalysis of C-C coupling reactions. A handful of L-Pd-Z examples exist that will be briefly introduced here with structures represented in Figure 1. The molecule [Me_2_P(CH_2_)_2_]_3_B was combined with Pd(PPh_3_)_4_ and led to formation of a complex (**1**) with tridentate coordination of the ambiphilic ligand to Pd(0) via phosphorus donor atoms; however the role of boron in stabilizing the Pd(0) center was difficult to establish without structural information [34]. Combination of Pd(OAc)_2_ with tris(2-mercapto-1-R-imidazolyl)hydroborate gave {[μ-κ^1^,κ^3^-B(mim^tBu^)_3_]Pd}_2_ (**2**) [35]. Treatment with PMe_3_ gave a monomeric [κ^4^-B(mim^tBu^)_3_]Pd(PMe_3_) complex (**3**). The dimeric complex **2** features Pd-B bond length of 2.073(4) Å and the monomeric complex **3** has Pd-B bond length of 2.050(8) Å. The triphosphine-borane ligand [*o*-*i*Pr_2_P-(C_6_H_4_)]_3_B (tpb) on combination with [Pd(P-*t*Bu_3_)_2_] led to formation of (tpb)Pd (**4**) that features a Pd-B bond length of 2.2535(17) Å [36]. The diphosphine-borane ligand [*o*-(*i*Pr_2_P)C_6_H_4_]_2_BPh (dpb) was combined with [PdCl_2_(COD)] to give [PdCl_2_(dpb)] (**5**) with Pd-B distance of 2.650(3) Å [37]. DFT calculations suggest very little donation of electron density from Pd(II)→B and significantly weaker bonding than found for the Pt(II)→B and Rh(I)→B interactions with the same ligand. It is noteworthy that the Pd-B distances in **3** and **4** are substantially shorter than in **5**, which is due primarily to the nucleophilicity of the metal. It can be agreed that in the absence of the Pd→B interaction, compounds **3** and **4** are d^10^ Pd(0) complexes whereas **5** is a d^8^ Pd(II) complex. However, it has been debated whether the Pd→B interaction should be considered as oxidative addition of the borane to Pd^n+^ to give Pd^n+2^ or if the oxidation state is unchanged on formation of the M-Z bond. This discrepancy is not real, and its origins have been described by Hill [38]. Both electron counting methods assume neat, whole numbers of electrons assigned to individual elements, which is an artificial abstraction to help understand chemical bonding. In actuality, as is most easily seen in molecular orbital theory, electron wavefunctions tend to be distributed over multiple atoms. The ambiguity of assigning formal charges and oxidation states as result of wavefunctions that span several atoms can be easily seen. To further illustrate, combination of 2,7-di-*tert*-butyl-5-diphenylboryl-4-diphenylphosphino-9,9-dimethylthioxanthene (TXPB) with [PdCl_2_(COD)] followed by reduction led to [Pd(TXPB)] (**6**). Rather than a direct Pd→B bond, complex **6** features an η^3^-BCC bonding mode with Pd-B distance of 2.320(5) Å [39]. In order to truly assess the degree of oxidation of the metal, one should use spectroscopic analyses such as near-edge energies of X-ray absorption spectra, X-ray photoelectron spectroscopy, or Mössbauer spectroscopy [40].

**Scheme 3:**
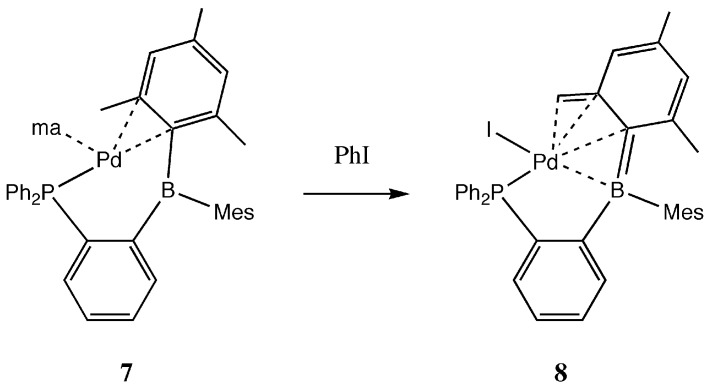
The [Ph_2_P(*o*-C_6_H_4_)BMes_2_]Pd(ma) complex **7** and the product **8** formed upon reaction with iodobenzene.

**Figure 2:**
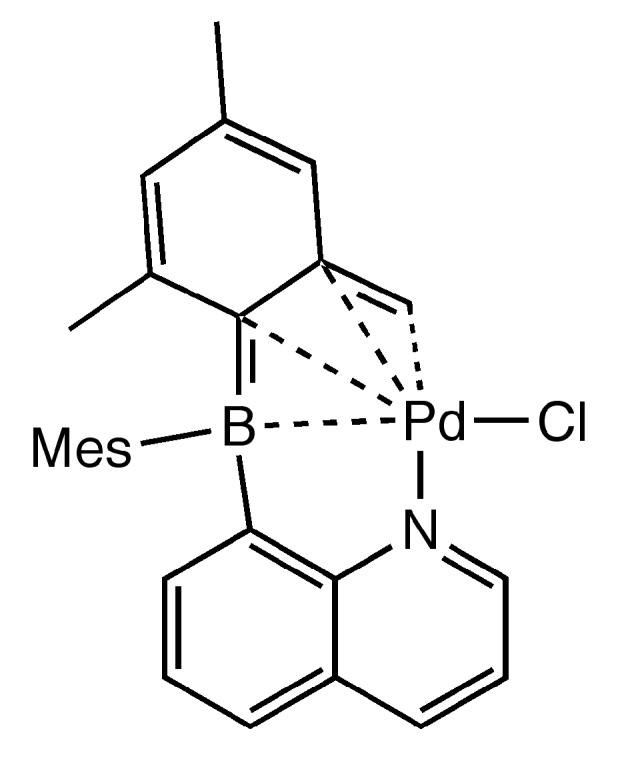
Chloro({2-[mesityl(quinolin-8-yl-κN)boryl]-3,5-dimethylphenyl}methyl-κC)-palladium(II) (**9**).

Recently, Bourissou and coworkers demonstrated Suzuki–Miyaura couplings were catalyzed by mixtures of Pd(OAc)_2_, [PdCl_2_(COD)], Pd_2_(dba)_3_, or [Pd(ma)(nbd)] (nbd = norborna-1,4-diene, ma = maleic anhydride) with Ph_2_P(*o*-C_6_H_4_)BMes_2_ [41,42]. Interestingly, the product formed from the reaction of [Pd(ma)(nbd)] with the monophosphine-borane was isolated and well characterized as [Ph_2_P(*o*-C_6_H_4_)BMes_2_]Pd(ma) (**7**) (Scheme 3). Single crystal X-ray data show Pd(0) within the cavity of Ph_2_P(*o*-C_6_H_4_)BMes_2_. However, instead of the FLP stabilizing Pd(0) via L-Pd-Z coordination, the structure of **7** consists of the monophosphine-borane participating as a bidentate chelating ligand to palladium via donation from the phosphine and η^2^-C-C coordination from the *ipso* and *ortho* carbons of a mesityl group. The preformed complex was shown to be a highly active Suzuki-Miyaura catalyst [41]. Interestingly, **7** was shown to oxidize in the presence of iodobenzene (Scheme 3) to form an η^4^-boratabutadiene iodophosphinopalladium(II) complex (**8**) [42]. The structure of **8** is very similar to that of **9** (Figure 2) reported by our group, which formed upon reaction of 8-(BMes_2_)quinoline with [PdCl_2_(PhCN)_2_] [43]. Cyclometalation occurs concomitantly with elimination of HCl to form a borapalladacycle in which the Pd(II) coordination sphere contains a quinolinyl nitrogen donor, chloride donor, and η^4^-boratabutadiene.

It was observed that **8** showed less activity in Suzuki-Miyaura coupling compared to **7**. Nevertheless, we were inspired to test the hypothesis that **9** is capable of catalyzing typical Pd-catalyzed C-C couplings. In this report, we present new findings that **9** does indeed show high activity in the Heck reaction.

## 2. Results and Discussion

**Scheme 4:**
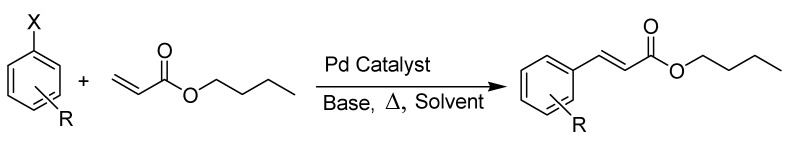
Coupling of arylhalides with *n*-butylacrylate.

Coupling of aryl halides with *n*-butylacrylate was studied (Scheme 4). The conditions of the reactions were empirically optimized upon comparison of activity in the presence of various bases and solvent systems. Very poor activity was observed with inorganic bases Cs_2_CO_3_, K_2_CO_3_, or NaOAc. The bulky organic base, Cy_2_NMe, also gave poor results. However, the reaction proceeded very well with *n*-Bu_3_N. The reactions were studied in the DMF/H_2_O solvent system. The best results (results using other bases/solvents are shown in the Supporting Information) were obtained in DMF without any addition of water; though, no steps were taken to remove water from the DMF reagent. The reactions were generally carried out at 140 °C. In these reaction conditions, the color of the solution was yellow and Pd black was not observed. The results of the coupling of various arylhalides with *n*-butylacrylate are summarized in Table 1.

The first entry in Table 1 shows that the coupling of iodobenzene with *n*-butylacrylate in the presence of 0.05 mol % **9** proceeded quite rapidly, as we isolated the coupled product in 88% yield after only 5 h reaction time. The second through fourth entries show that the catalyst loading could be reduced substantially in order to demonstrate the catalyst turnover number (TON). At 0.001 mol % loading of **9**, after 20 h of reaction time a 90% isolated yield of the coupled product was found that corresponds to a TON ~ 90,000. Potentially this figure could be improved with further optimization; though we already show that the catalyst is capable of TON comparable with active Pd-catalysts reported in the literature. For example, a highly active Pd-PCP pincer complex achieved a TON of 143,000 for the coupling of iodobenzene with *n*-butylacrylate [44]. A heterogeneous catalyst consisting of Pd encapusalated within cross-linked poly(1,3-diethynylbenzene) gave TON ~ 100,000 [45]. For additional comparison, combination of Pd(OAc)_2_ with a trifunctional *N*,*N*,*O*-terdentate amido/pyridyl/carboxylate ligand produced a catalyst that achieved TON ~ 10,000 for coupling of iodobenzene with *n*-butylacrylate [46]. We found that combination of PdCl_2_ with two equivalents of quinoline at 0.01 mol % Pd catalyzed the coupling of iodobenzene with *n*-butylacrylate with TON ~ 9300, and this result is similar to the initial screening of Pd(quinoline-8-carboxylate)_2_ at 0.01 mol % loading that gave a TON ~ 8700 [47]. However, with respect to achieving high TON [48] combinations of Pd(OAc)_2_ with phosphines has been shown to give TON ~ 10^6^ for the coupling of *p*-bromoacetophenone with *n*-butylacrylate [49]. Ferrocenylimine palladacycles were shown to achieve TON ~ 3.6 × 10^6^ [50]. Imine-palladacycle catalysts were shown to reach TON ~ 1.4 × 10^6^ for the coupling of iodobenzene with methylacrylate [51]. Oxime-palladacycle catalysts were shown to reach TON ~ 10^10^ for the coupling of iodobenzene with alkylacrylate [52,53] Catalyst **9** was completely ineffective for the coupling of bromobenzene with *n*-butylacrylate; however, *p*-cyanobromobenzene could be utilized as a substrate. At 0.01 mol % loading of **9**, the yield was already limited to 58%, corresponding to TON ~ 5800. For comparison, it was shown that mixture of Pd(OAc)_2_ with phosphines could catalyze coupling of arylbromides with *n*-butylacrylate with TON ~ 10^6^ [49]. A comparison of the coupling of *p*-R-C_6_H_4_I (R = OCH_3_, H, NO_2_) with **9** as catalyst shows the tolerance for the functional groups; however, there was not a clear indication of an electronic effect of the substrate on the rate of the reaction.

**Table 1:** Heck coupling reaction of aryl halides with *n*-butylacrylate using **9** as catalyst and comparison with other data from the literature. ^*a*^ Isolated yield (our work).

Catalyst	X	R	mol % Catalyst	T (°C)	Time/h	Yield (%) ^*a*^	TON
**9**	I	H	0.05	140	5	88	1800
**9**	I	H	0.01	140	5	94	9400
**9**	I	H	0.005	140	10	89	18,000
**9**	I	H	0.001	140	20	90	90,000
**9**	I	*p*-NO_2_	0.01	140	10	83	8300
**9**	I	*p*-OCH_3_	0.01	140	10	79	7900
**9**	Br	*p*-CN	0.01	140	10	58	5800
**9**	Br	H	0.01	140	5	0	0
Quinoline:PdCl_2_ = 2:1	I	H	0.01	140	5	93	9300
Pd(OAc)_2_ [54]	I	H	0.5	80	2	96	192
PR_3_:Pd(OAc)_2_ = 2:1 [49]	Br	*p*-C(O)CH_3_	0.0001	130	24	100	10^6^
Ref. [47]	I	H	0.01	130	30	87	8700
Ref. [46]	I	H	0.01	145	20	95	9500
Ref. [46]	Br	*p*-OCH_3_	0.01	145	20	93	9300
Ref. [44]	I	H	0.0007	160	14	100	1.4 × 10^5^
Pd@PDEB [45]	I	H	0.001	100	48	100	10^5^
Ref. [50]	I	H	2.73 × 10^−5^	100	145	99	3.6 × 10^6^
Ref. [52]	I	H	10^−8^	160	72	98	10^10^

**Scheme 5:**
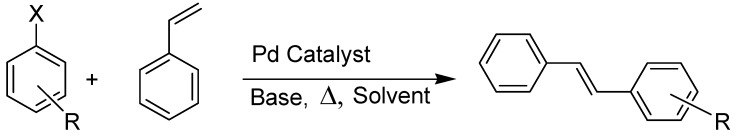
Coupling of arylhalide with styrene.

Coupling of aryl halides with styrene was studied as well (Scheme 5). Again, the reaction proceeded poorly in the presence of inorganic bases Cs_2_CO_3_, K_2_CO_3_, NaOAc, or with the bulky organic base Cy_2_NMe; whereas, the *n*-Bu_3_N gave good activity. The reactions proceeded smoothly in DMF, and addition of water led to substantially lower activity. Again, the solutions remained yellow and homogeneous throughout the reaction and Pd black was not observed. The results of the coupling of various aryl halides with styrene are summarized in Table 2.

**Table 2:** Heck coupling reaction of aryl halides with styrene using **9** as catalyst and comparison with other data from the literature. ^*a*^ Isolated yield (our work).

Catalyst	X	R	mol % Catalyst	T (°C)	Time/h	Yield (%) ^*a*^	TON
**9**	I	H	0.01	140	5	20	2000
**9**	I	H	0.01	140	10	37	3700
**9**	I	*p*-OCH_3_	0.01	140	10	42	4200
**9**	Br	*p*-CN	0.01	140	10	30	3000
**9**	Br	H	0.01	140	5	0	0
PPh_3_:Pd(OAc)_2_ = 2:1 [49]	Br	*p*-C(O)CH_3_	0.0001	130	72	94	940,000
Ref. [46]	I	H	0.01	145	20	96	9600
Ref. [46]	Br	*p*-OCH_3_	0.01	145	20	95	9500
Ref. [44]	I	H	0.0007	140	60	93	133,000
Ref. [52]	Br	*p*-OCH_3_	0.001	160	16	97	97,000
Ref. [51]	I	H	0.0007	140	80	74	106,000
Ref. [55]	Br	*p*-CHO	1	80	15	95	95
Ref. [56]	I	*p*-OCH_3_	2	120	8	94	47
Ref. [57]	Br	*p*-C(O)CH_3_	0.00001	180	69	57.5	5,750,000
Ref. [58]	Br	H	0.005	140	1	83	16,600

The reactions with styrene were substantially slower than comparable reactions with *n*-butylacrylate. Interestingly, we again observe no reaction with bromobenzene yet an appreciable reaction with *p*-cyanobromobenzene. The activity of **9** for coupling of styrene to aryl halides appears to be markedly lower than other catalysts reported in the literature. Even a 2:1 mixture of PPh_3_ and Pd(OAc)_2_ has been shown to catalyze coupling of styrene to *p*-bromoacetophenone with TON ~ 9.4 × 10^5^ [49]. Oxime-palladacycle catalysts were shown to reach TON ~ 10^5^ for the coupling of *p*-bromoanisole with styrene [52]. Imine-palladacycle catalysts were shown to reach TON ~ 10^5^ for the coupling of iodobenzene with styrene [51]. A Pd-PCP pincer complex achieved a TON of 133,000 for the coupling of iodobenzene with styrene [44]. An orthopalladated triarylphosphite catalyst achieved TON ~ 5.75 × 10^6^ for the coupling of *p*-bromoacetophenone with styrene [57].

**Scheme 6:**
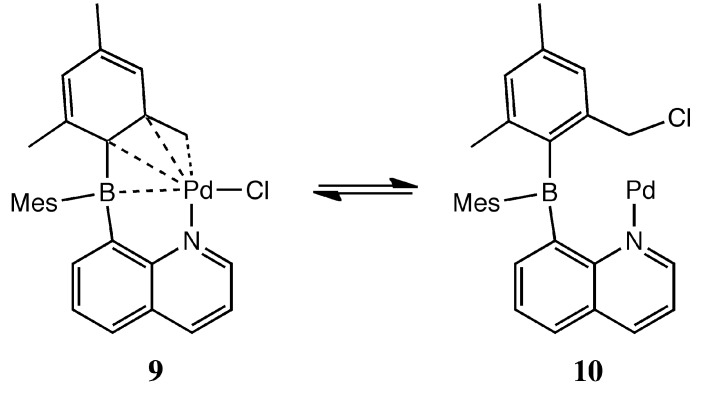
Postulated molecular catalyst **10** involved in the oxidative addition step.

Palladacycles have received much attention as catalysts for C-C coupling reactions [48,49,59,60]. The palladacycle must undergo a chemical change in order to generate the catalytically active Pd(0) species. Hartwig postulated a mechanism that involved a reductive elimination step to form a classical phosphine stabilized Pd(0) species [61]. Conceivably, the palladacycle **9** could undergo reductive elimination of the tucked mesityl and chloride ligands to form a postulated catalyst **10** (Scheme 6). The observation of Heck reactions catalyzed by compound **9** suggests this possibility; however so far we were unable to obtain structural or spectroscopic evidence for this hypothesized intermediate. Such information would be helpful in understanding the catalyst reactivity, for example, whether the Pd(0) stabilization in **10** resembles complex **7** or an example of a bent L-Pd(0)-Z complex. In either case the Pd(0) would be coordinatively unsaturated and have a bent geometry, both of which would enhance the reactivity for oxidative addition reactions. It is important to point out that **8** was reported to be less active than **7** for Suzuki couplings. So far, we could not compare the reactivity of **9** and **10**, since we have not found a way to isolate **10**.

Remarkably, the catalyst **9** was stable in the reaction conditions of elevated temperature for extended time periods without formation of any palladium black precipitate. The reaction solution is a yellow color throughout the entire course of the reaction. Additionally, we note that the onset of catalytic activity is immediate without any induction period. These observations suggest that the catalyst species is molecular rather than a colloidal nanoparticle catalyst.

## 3. Experimental Section

### 3.1. General Information

Compound **9** was prepared according to the literature [43]. All organic reagents and solvents were obtained from commercial sources and used without further purification. A GCMS-QP2010SE gas chromatograph-mass spectrometer (Shimadzu Corp., Kyoto, Japan) was used for GCMS analyses. NMR spectra were recorded on an Avance 400 MHz spectrometer (Bruker, Billerica, MA, USA).

### 3.2. Heck Cross Coupling Reaction

A 50 mL flask was charged with 1 mmol aryl halide, 1.2 mmol olefin, 2 mmol *n*-Bu_3_N, **9**, and DMF (5 mL). The reaction mixture was then stirred at 140 °C h under N_2_. The reaction was worked up by extraction with ether and washing with DI H_2_O. The organic phase was collected and dried over anhydrous sodium sulfate. The residue was purified by flash column chromatography to afford the desired product. NMR spectra of isolated products matched well with the literature [62].

## 4. Conclusions

To summarize, the molecule chloro({2-[mesityl(quinolin-8-yl-κN)boryl]-3,5-dimethylphenyl}-methyl-κC)palladium(II) was demonstrated to catalyze Heck reactions of *n*-butylacrylate or styrene with aryl halides. Turnover numbers in the coupling of aryliodides with *n*-butylacrylate were quite high; however, this was not the case with styrene. We speculate, based on discussion in the chemical literature, that the palladacycle **9** undergoes reductive elimination to form active Pd(0) species. The postulated active catalyst species could potentially be a bent L-Pd(0)-Z species; however, structural or spectroscopic evidence has not been obtained so far. Further work is needed to elucidate the reactive catalyst species, and to determine whether this motif offers advantages in catalysis. The results so far suggest that the preparation of pre-formed bent L-Pd(0)-Z catalysts may be a fruitful avenue for development of active catalyst materials.

## Supplementary Materials

Supplementary materials can be accessed at: http://www.mdpi.com/1420-3049/20/07/12979/s1.

## References

[1] Li H., Johansson Seechurn C.C.C., Colacot T.J. (2012). Development of Preformed Pd Catalysts for Cross-Coupling Reactions, Beyond the 2010 Nobel Prize. ACS Catal..

[2] Beletskaya I.P., Cheprakov A.V. (2000). The Heck Reaction as a Sharpening Stone of Palladium Catalysis. Chem. Rev..

[3] Cabri W., Candiani I. (1995). Recent Developments and New Perspectives in the Heck Reaction. Acc. Chem. Res..

[4] Ozawa F., Kubo A., Hayashi T. (1992). Generation of tertiary phosphine-coordinated Pd(0) species from Pd(OAc)_2_ in the catalytic Heck reaction. Chem. Lett..

[5] Littke A.F., Fu G.C. (1999). Heck Reactions in the Presence of P(*t*-Bu)_3_: Expanded Scope and Milder Reaction Conditions for the Coupling of Aryl Chlorides. J. Org. Chem..

[6] Fu G.C. (2008). The Development of Versatile Methods for Palladium-Catalyzed Coupling Reactions of Aryl Electrophiles through the Use of P(*t*-Bu)_3_ and PCy_3_ as Ligands. Acc. Chem. Res..

[B7-molecules-20-12979] 7.We note that numerous supported Pd(0) catalysts, such as Pd/C or zeolite supported Pd, exist and that these are phosphine-free.

[8] Selvakumar K., Zapf A., Beller M. (2002). New Palladium Carbene Catalysts for the Heck Reaction of Aryl Chlorides in Ionic Liquids. Org. Lett..

[9] Park S.B., Alper H. (2003). Highly Efficient, Recyclable Pd(II) Catalysts with Bisimidazole Ligands for the Heck Reaction in Ionic Liquids. Org. Lett..

[10] Consorti C.S., Zanini M.L., Leal S., Ebeling G., Dupont J. (2003). Chloropalladated Propargyl Amine: A Highly Efficient Phosphine-Free Catalyst Precursor for the Heck Reaction. Org. Lett..

[11] Beletskaya I.P., Kashin A.N., Karlstedt N.B., Mitin A.V., Cheprakov A.V., Kazankov G.M. (2001). NC-palladacycles as highly effective cheap precursors for the phosphine-free Heck reactions. J. Organomet. Chem..

[12] Mino T., Shirae Y., Sasai Y., Sakamoto M., Fujita T. (2006). Phosphine-free palladium catalyzed Mizoroki-Heck reaction using hydrazone as a ligand. J. Org. Chem..

[13] Gruber A.S., Pozebon D., Monteiro A.L., Dupont J. (2001). On the use of phosphine-free PdCl_2_(SEt_2_)_2_ complex as catalyst precursor for the Heck reaction. Tetrahedron Lett..

[14] Herrman W.A., Reisinger C.P., Spiegler M. (1998). Chelating *N*-heterocyclic carbene ligands in palladium-catalyzed heck-type reactions. J. Organomet. Chem..

[15] Joost M., Zeineddine A., Estevez L., Mallet-Ladeira S., Miqueu K., Amgoune A., Bourissou D. (2014). Facile Oxidative Addition of Aryl Iodides to Gold(I) by Ligand Design: Bending Turns on Reactivity. J. Am. Chem. Soc..

[16] Stephan D.W., Erker G. (2010). Frustrated Lewis Pairs: Metal-free Hydrogen Activation and More. Angew. Chem. Int. Ed..

[17] Stephan D.W. (2009). Frustrated Lewis pairs: A new strategy to small molecule activation and hydrogenation catalysis. Dalton Trans..

[18] Rokob T.A., Hamza A., Papai I. (2009). Rationalizing the Reactivity of Frustrated Lewis Pairs: Thermodynamics of H_2_ Activation and the Role of Acid-Base Properties. J. Am. Chem. Soc..

[19] Welch G.C., San Juan R.R., Masuda J.D., Stephan D.W. (2006). Reversible, Metal-Free Hydrogen Activation. Science.

[20] Welch G.C., Stephan D.W. (2007). Facile Heterolytic Cleavage of Dihydrogen by Phosphines and Boranes. J. Am. Chem. Soc..

[21] Momming C.M., Otten E., Kehr G., Frohlich R., Grimme S., Stephan D.W., Erker G. (2009). Reversible Metal-Free Carbon Dioxide Binding by Frustrated Lewis Pairs. Angew. Chem. Int. Ed..

[22] Berkefeld A., Piers W.E., Parvez M. (2010). Tandem Frustrated Lewis Pair/Tris(pentafluorophenyl)borane-Catalyzed Deoxygenative Hydrosilylation of Carbon Dioxide. J. Am. Chem. Soc..

[23] Ashley A.E., Thompson A.L., O’Hare D. (2009). Non-Metal-Mediated Homogeneous Hydrogenation of CO_2_ to CH_3_OH. Angew. Chem. Int. Ed..

[24] Appelt C., Westenberg H., Bertini F., Ehlers A.W., Slootweg J.C., Lammertsma K., Uhl W. (2011). Geminal Phosphorus/Aluminum-Based Frustrated Lewis Pairs: C-H *versus* C≡C Activation and CO_2_ Fixation. Angew. Chem. Int. Ed..

[25] Lu Z., Wang Y., Liu J., Lin Y.J., Li Z.H., Wang H. (2013). Synthesis and Reactivity of the CO_2_ Adducts of Amine/Bis(2,4,6-tris(trifluoromethyl)phenyl)borane Pairs. Organometallics.

[26] Rochette E., Courtemanche M.A., Pulis A.P., Bi W., Fontaine F.G. (2015). Ambiphilic Frustrated Lewis Pair Exhibiting High Robustness and Reversible Water Activation: Towards the Metal-Free Hydrogenation of Carbon Dioxide. Molecules.

[27] Roesler R., Piers W.E., Parvez M. (2003). Synthesis, structural characterization and reactivity of the aminoborane 1-(NPh_2_)-2-[B(C_6_F_5_)_2_]C_6_H_4_. J. Organomet. Chem..

[28] Fromel S., Frohlich R., Daniliuc C.G., Kehr G., Erker G. (2012). Halogen Addition to a Frustrated Lewis Pair. Eur. J. Inorg. Chem..

[29] Son J.H., Tamang S.R., Hoefelmeyer J.D. (2015). Bis(3-bromomesityl)8-quinolyliniumboron(III) tribromide. Acta Cryst..

[30] Sircoglou M., Bouhadir G., Saffon N., Miqueu K., Bourissou D. (2008). A Zwitterionic Gold(I) Complex from an Ambiphilic Diphosphino–Alane Ligand. Organometallics.

[31] Dureen M.A., Welch G.C., Gilbert T.M., Stephan D.W. (2009). Heterolytic Cleavage of Disulfides by Frustrated Lewis Pairs. Inorg. Chem..

[32] Fontaine F.G., Boudreau J., Thibault M.H. (2008). Coordination Chemistry of Neutral (L_n_)-Z Amphoteric and Ambiphilic Ligands. Eur. J. Inorg. Chem..

[33] Amgoune A., Bourissou D. (2011). σ-Acceptor, Z-type ligands for transition metals. Chem. Commun..

[34] Grobe J., Lutke-Brochtrup K., Krebs B., Lage M., Niemeyer H.H., Wurthwein E.U. (2006). Alternative Ligands, XXXVII. Phosphane Ligands with Boron as Lewis-acidic Centre: Synthesis and Coordinating Properties. Z. Naturforsch. B.

[35] Pang K., Quan S.M., Parkin G. (2006). Palladium complexes with Pd→B dative bonds: Analysis of the bonding in the palladaboratrane compound [κ^4^B(mim^But^)_3_]Pd(PMe_3_). Chem. Commun..

[36] Sircoglou M., Bontemps S., Bouhadir G., Saffon N., Miqueu K., Gu W., Mercy M., Chen C.H., Foxman B.M., Maron L. (2008). Group 10 and 11 Metal Boratranes (Ni, Pd, Pt, CuCl, AgCl, AuCl, and Au^+^) Derived from a Triphosphine–Borane. J. Am. Chem. Soc..

[37] Bontemps S., Sircoglou M., Bouhadir G., Puschmann H., Howard J.A.K., Dyer P.W., Miqueu K., Bourissou D. (2008). Ambiphilic Diphosphine–Borane Ligands: Metal→Borane Interactions within Isoelectronic Complexes of Rhodium, Platinum and Palladium. Chem. Eur. J..

[38] Hill A.F. (2006). An Unambiguous Electron-Counting Notation for Metallaboratranes. Organometallics.

[39] Emslie D.J.H., Harrington L.E., Jenkins H.A., Robertson C.M., Britten J.F. (2008). Group 10 Transition-Metal Complexes of an Ambiphilic PSB-Ligand: Investigations into η^3^(*BCC*)-Triarylborane Coordination. Organometallics.

[40] Sircoglou M., Bontemps S., Mercy M., Saffon N., Takahashi M., Bouhadir G., Maron L., Bourissou D. (2007). Transition-Metal Complexes Featuring Z-Type Ligands: Agreement or Discrepancy between Geometry and d^n^ Configuration?. Angew. Chem. Int. Ed..

[41] Malacea R., Saffon N., Gomez M., Bourissou D. (2011). A new insight into *ortho*-(dimesitylboryl)diphenylphosphines: Applications in Pd-catalyzed Suzuki–Miyaura couplings and evidence for secondary π-interaction. Chem. Commun..

[42] Malacea R., Chahdoura F., Devillard M., Saffon N., Gomez M., Bourissou D. (2013). *ortho*-(Dimesitylboryl)phenylphosphines: Positive Boryl Effect in the Palladium-Catalyzed Suzuki–Miyaura Coupling of 2-Chloropyridines. Adv. Synth. Catal..

[43] Son J.H., Pudenz M.A., Hoefelmeyer J.D. (2010). Reactivity of the Bifunctional Ambiphilic Molecule 8-(dimesitylboryl)quinoline: Hydrolysis and Coordination to Cu(I), Ag(I) and Pd(II). Dalton Trans..

[44] Ohff M., Ohff A., van der Boom M.E., Milstein D. (1997). Highly Active Pd(II) PCP-Type Catalysts for the Heck Reaction. J. Am. Chem. Soc..

[45] Dong Z., Ye Z. (2014). Reusable, Highly Active Heterogeneous Palladium Catalyst by Convenient Self-Encapsulation Cross-Linking Polymerization for Multiple Carbon—Carbon Cross-Coupling Reactions at ppm to ppb Palladium Loadings. Adv. Synth. Catal..

[46] Kantam M.L., Srinivas P., Yadav J., Likhar P.R., Bhargava S. (2009). Trifunctional *N*,*N*,*O*-Terdentate Amido/Pyridyl Carboxylate Ligated Pd(II) Complexes for Heck and Suzuki Reactions. J. Org. Chem..

[47] Cui X., Li J., Zhang Z.P., Fu Y., Liu L., Guo Q.X. (2007). Pd(quinoline-8-carboxylate)_2_ as a Low-Priced, Phosphine-Free Catalyst for Heck and Suzuki Reactions. J. Org. Chem..

[48] Farina V. (2004). High-Turnover Palladium Catalysts in Cross-Coupling and Heck Chemistry: A Critical Overview. Adv. Synth. Catal..

[49] Herrman W.A., Brossmer C., Reisinger C.P., Riermeier T.H., Ofele K., Beller M. (1997). Palladacycles: Efficient New Catalysts for the Heck Vinylation of Aryl Halides. Chem. Eur. J..

[50] Wu Y., Hou J., Yun H., Cui X., Yuan R. (2001). Cyclopalladated ferrocenylimines: Highly active catalysts for Heck reactions. J. Organomet. Chem..

[51] Ohff M., Ohff A., Milstein D. (1999). Highly active Pd^II^ cyclometallated imine catalysts for the Heck reaction. Chem. Commun..

[52] Alonso D.A., Najera C., Pacheco M.C. (2002). Oxime-Derived Palladium Complexes as Very Efficient Catalysts for the Heck-Mizoroki Reaction. Adv. Synth. Catal..

[53] Alonso D.A., Najera C., Pacheco M.C. (2000). Oxime Palladacycles: Stable and Efficient Catalysts for Carbon–Carbon Coupling Reactions. Org. Lett..

[54] Amini M., Bagherzadeh M., Moradi-Shoeili Z., Boghaei D.M. (2012). Pd(OAc)_2_ without added ligand as an active catalyst for Mizoroki–Heck reaction in aqueous media. RSC Adv..

[55] Özdemir I., Yiğit M., Çetinkaya E., Çetinkaya B. (2006). Synthesis of novel palladium *N*-heterocyclic-carbene complexes as catalysts for Heck and Suzuki cross-coupling reactions. Appl. Organomet. Chem..

[56] Wang R., Twamley B., Shreeve J.M. (2005). A Highly Efficient, Recyclable Catalyst for C–C Coupling Reactions in Ionic Liquids: Pyrazolyl-Functionalized *N*-Heterocyclic Carbene Complex of Palladium(II). J. Org. Chem..

[57] Albisson D.A., Bedford R.B., Scully P.N. (1998). Orthopalladated triarylphosphite complexes as highly efficient catalysts in the Heck reaction. Tetrahedron Lett..

[58] Heidenreich R.G., Köhler K., Krauter J.G.E., Pietsch J. (2002). Pd/C as a Highly Active Catalyst for Heck, Suzuki and Sonogashira Reactions. Synlett.

[59] Herrman W.A., Bohm V.P.W., Reisinger C.P. (1999). Application of palladacycles in Heck type reactions. J. Organomet. Chem..

[60] Dupont J., Consorti C.S., Spencer J. (2005). The Potential of Palladacycles: More than Just Precatalysts. Chem. Rev..

[61] Louie J., Hartwig J.F. (1996). A Route to Pd^0^ from Pd^II^ Metallacycles in Amination and Cross-Coupling Chemistry. Angew. Chem. Int. Ed..

[62] Huang S.H., Chen J.R., Tsai F.Y. (2010). Palladium(II)/Cationic 2,2′-Bipyridyl System as Highly Efficient and Reusable Catalyst for the Mizoroki-Heck Reaction in Water. Molecules.

