# Supplementary material for: Chloro({2-[mesityl(quinolin-8-yl-κN)boryl]-3,5-dimethyl-phenyl}methyl-κC)palladium(II) as a Catalyst for Heck Reactions
Source: Molecules. 2015 Jul 17;20(7):12979–91. doi: 10.3390/molecules200712979 (PMC6332355; doi:10.3390/molecules200712979)
Supplement: Supplementary file 1 [file molecules-20-12979-s001.pdf]

Supporting Information

Contents

Table S1. Heck coupling reactions of aryl halides and *n*-butylacrylate with different bases S2
Table S2. Heck coupling reactions of aryl halide and styrene with different bases S2
Experimental...S3
Figure S1. ^{1}H-NMR of 3-phenylacrylic acid *n*-butyl ester...S4
Figure S2. ^{1}H-NMR of 3-(4-nitrophenyl)acrylic acid *n*-butyl ester. S5
Figure S3. ^{1}H-NMR of 3-(4-cyanophenyl)acrylic acid *n*-butyl ester. S6
Figure S4. ^{1}H-NMR of 3-(4-methoxyphenyl)acrylic acid *n*-butyl ester......................................S7
Figure S5. ^{1}H-NMR of stilbene. S8
Figure S6. ^{1}H NMR of 4-methoxystilbene. S9
Figure S7. ^{1}H NMR of 4-cyanostilbene. S10
Figure S8. GC-MS data of 3-phenylacrylic acid *n*-butyl ester.. ....S11
Figure S9. GC-MS data of 3-(4-methoxyphenyl)acrylic acid *n*-butyl ester.. ....S12
Figure S10. GC-MS data of 3-(4-cyanophenyl)acrylic acid *n*-butyl ester.. ....S13
Figure S11. GC-MS data of 3-(4-nitrophenyl)acrylic acid *n*-butyl ester.. ....S14
Figure S12. GC-MS data of 4-methoxystilbene.. ....S15
Figure S13. GC-MS data of stilbene.. ....S16
Figure S14. GC-MS data of 4-cyanostilbene.. ....S17

Table S1. Heck coupling reaction of aryl halides with *n*-butylacrylate with different solvents and bases using 0.005 mol % catalyst.

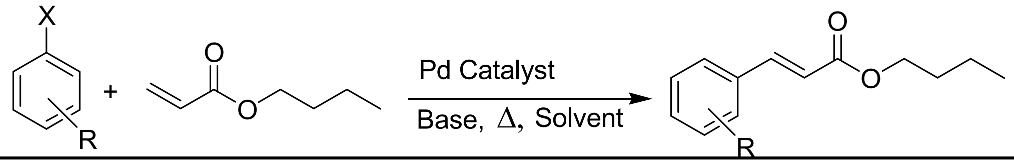

Entry	X	R	Solvent	Base	Time/h	Temp/°C	Yield(%) [b]
1	I	H	DMF	Cs_2CO_3	20	140	0
2	Br	H	DMF	Cs_2CO_3	20	140	0
3	Br	*m*-MeO	DMF	Cs_2CO_3	20	140	0
4	Br	*o*-MeO	DMF	Cs_2CO_3	20	140	0
5	Br	*m*-Me	DMF	Cs_2CO_3	20	140	0
6	Br	*m*-Cl	DMF	Cs_2CO_3	20	140	0
7	I	H	DMF/H_2O *	Cs_2CO_3	5	145	0
8	I	H	MeOH	$LiOH·H_2O$	5	65	0
9	Br	H	DMF	Cy_2NMe	15	140	0
10	Br	H	NMP [+]	Cs_2CO_3	12	100	0
11	I	H	DMF/H_2O *	Cs_2CO_3	14	145	0
12	I	H	DMF	Cy_2NMe	5	145	0
13	I [a]	H	DMF	NBu_3	5	140	96

[a] reaction with 0.05 mol % catalyst; [b] GC-MS yield; * Solvent mixture was in 4 (DMF):1 (H_2O) ratio; [+] *N*-Methyl-2-pyrrolidone.

Table S2. Heck coupling reaction of aryl halides with styrene with different bases using 0.005 mol % catalyst.

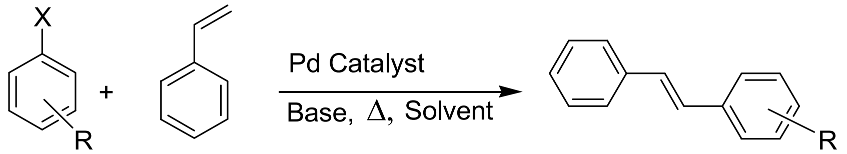

Entry	X	R	DMF/H_2O (*v*/*v*)	Base	Time/h	Temp/°C	Yield(%) [c]
**1**	I	H	DMF	Cs_2CO_3	20	140	33
**2**	I	H	4:1	Cs_2CO_3	4	140	51
**3**	Br	H	4:1	Cs_2CO_3	4	140	0
**4**	Br	*m*-Cl	4:1	Cs_2CO_3	4	140	0
**5**	Br	*m*-MeO	4:1	Cs_2CO_3	4	140	0
**6**	Br	H	4:1	Cs_2CO_3	12	80	0
**7**	Br	*m*-MeO	DMF	K_2CO_3	12	70	0
**8**	Br	H	DMF	NaOAc	12	100	0
**9**	Br [a]	H	4:1	Cs_2CO_3	14	140	0
**10**	Br	H	4:1	Cy_2NMe	15	140	0
**11**	I	*p*-NO_2	7:1	Cs_2CO_3	4	150	0
**12**	I	*p*-MeO	4:1	Cs_2CO_3	4	150	0
**13**	I [b]	H	DMF	NBu_3	5	140	55

[a] reaction with 0.05 mol % catalyst; [b] reaction with 0.01 mol % catalyst; [c] GC-MS yield.

Experimental

3-Phenylacrylic acid n-butyl ester (**1a**). Yellow oil. ^{1}H-NMR: δ 0.97 (t, *J* = 7.4 Hz, 3H), 1.40–1.49 (m, 2H), 1.66–1.73 (m, 2H), 4.21 (t, *J* = 6.8 Hz, 2H), 6.44 (d, *J* = 8.0 Hz, 1H), 7.37–7.39(m, 3H), 7.51–7.54 (m, 2H), 7.68 (d, *J* = 16.4 Hz, 1H)

3-(4-Nitrophenyl)acrylic acid n-butyl ester (**1b**). Yellow solid. ^{1}H-NMR: δ 0.97 (t, *J* = 7.4 Hz, 3H), 1.40–1.49 (m, 2H), 1.67–1.74 (m, 2H), 4.24 (t, *J* = 6.8 Hz, 2H), 6.56 (d, *J* = 16.0 Hz, 1H), 7.66–7.72 (m, 3H), 8.25 (d, *J* = 9.2 Hz, 2H)

3-(4-Cyanophenyl)acrylic acid n-butyl ester (**1c**). Yellow oil. ^{1}H-NMR: δ 0.96 (t, *J* = 7.4 Hz, 3H), 1.39–1.48 (m, 2H), 1.66–1.71 (m, 2H), 4.23 (t, *J* = 6.8 Hz, 2H), 6.52 (d, *J* = 16 Hz, 1H), 7.60–7.69 (m, 5H)

3-(4-Methoxyphenyl)acrylic acid n-butyl ester (**1d**). Yellow oil. ^{1}H-NMR: δ 0.96 (t, *J* = 7.4 Hz, 3H), 1.39–1.48 (m, 2H), 1.65–1.72 (m, 2H), 3.84 (s, 3H), 4.20 (t, *J* = 6.7 Hz, 2H), 6.31 (d, *J* = 16.0 Hz, 1H), 6.90 (d, *J* = 8.8 Hz, 2H), 7.48 (d, *J* = 8.6 Hz, 2H), 7.64 (d, *J* = 16.0 Hz, 1H)

Stilbene (**1e**). Colorless solid. ^{1}H-NMR: δ 7.12 (s, 2H), 7.29 (t, *J* = 6.6 Hz, 2H), 7.36(t, *J* = 7.6 Hz, 4H), 7.52 (dd, *J* = 8.2 Hz, 4H)

4-Methoxystilbene (**1f**). Colorless solid. ^{1}H-NMR: δ 3.83 (s, 3H), 6.90 (d, *J* = 8.8 Hz, 2H), 6.98 (d, *J* = 16.3 Hz, 1H), 7.07 (d, *J* = 16.3 Hz, 1H), 7.21–7.25 (m, 1H), 7.35 (t, *J* = 7.6 Hz, 2H), 7.44–7.50 (m, 4H)

4-Cyanostilbene (**1g**). Colorless solid. ^{1}H-NMR: δ 7.09 (d, *J* = 16.3 Hz, 1H), 7.22 (d, *J* = 16.3 Hz, 1H), 7.30–7.34 (m, 1H), 7.39 (t, *J* = 7.5 Hz, 1H), 7.52–7.65 (m, 6H)

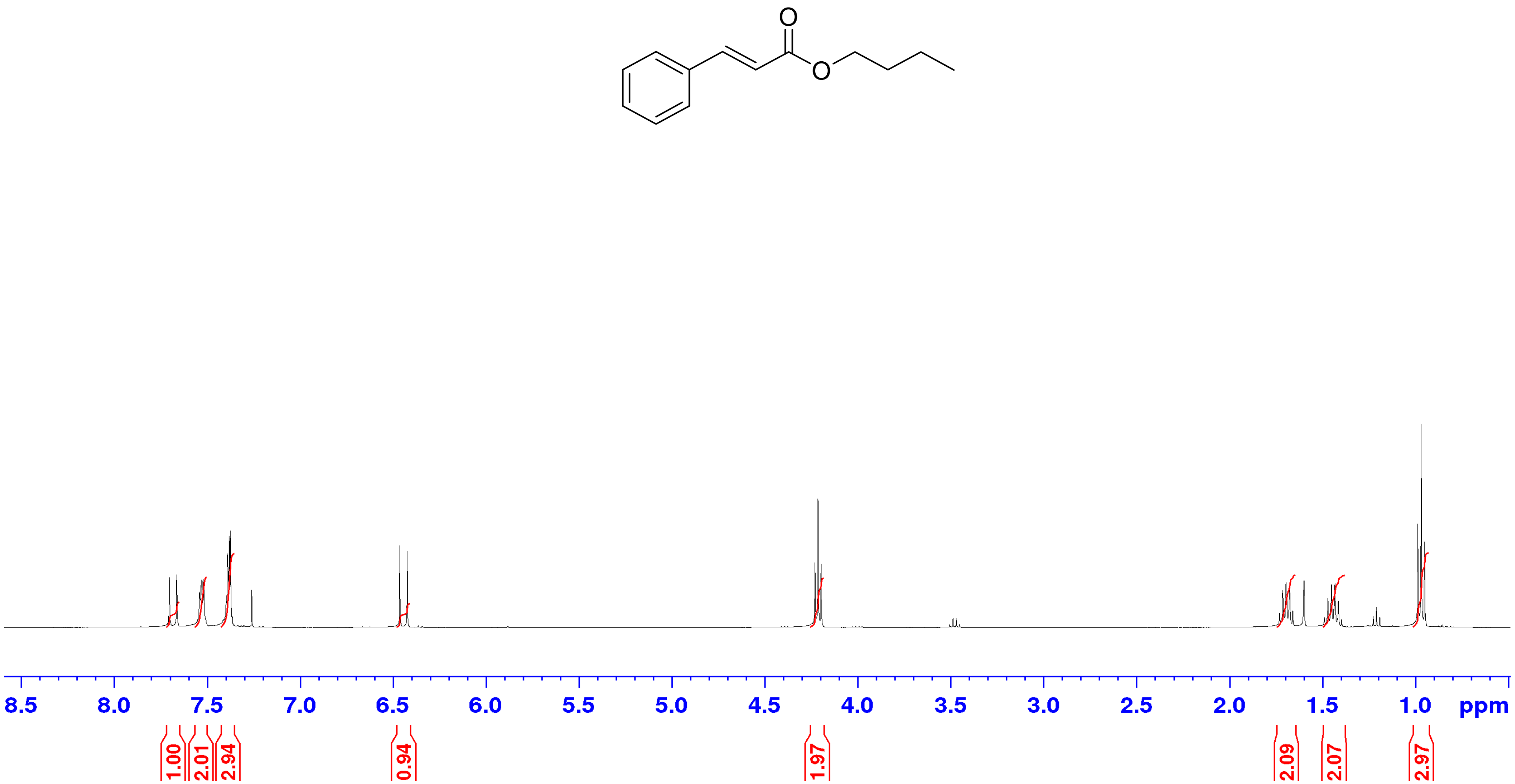

Figure S1. ^{1}H-NMR of 3-phenylacrylic acid *n*-butyl ester.

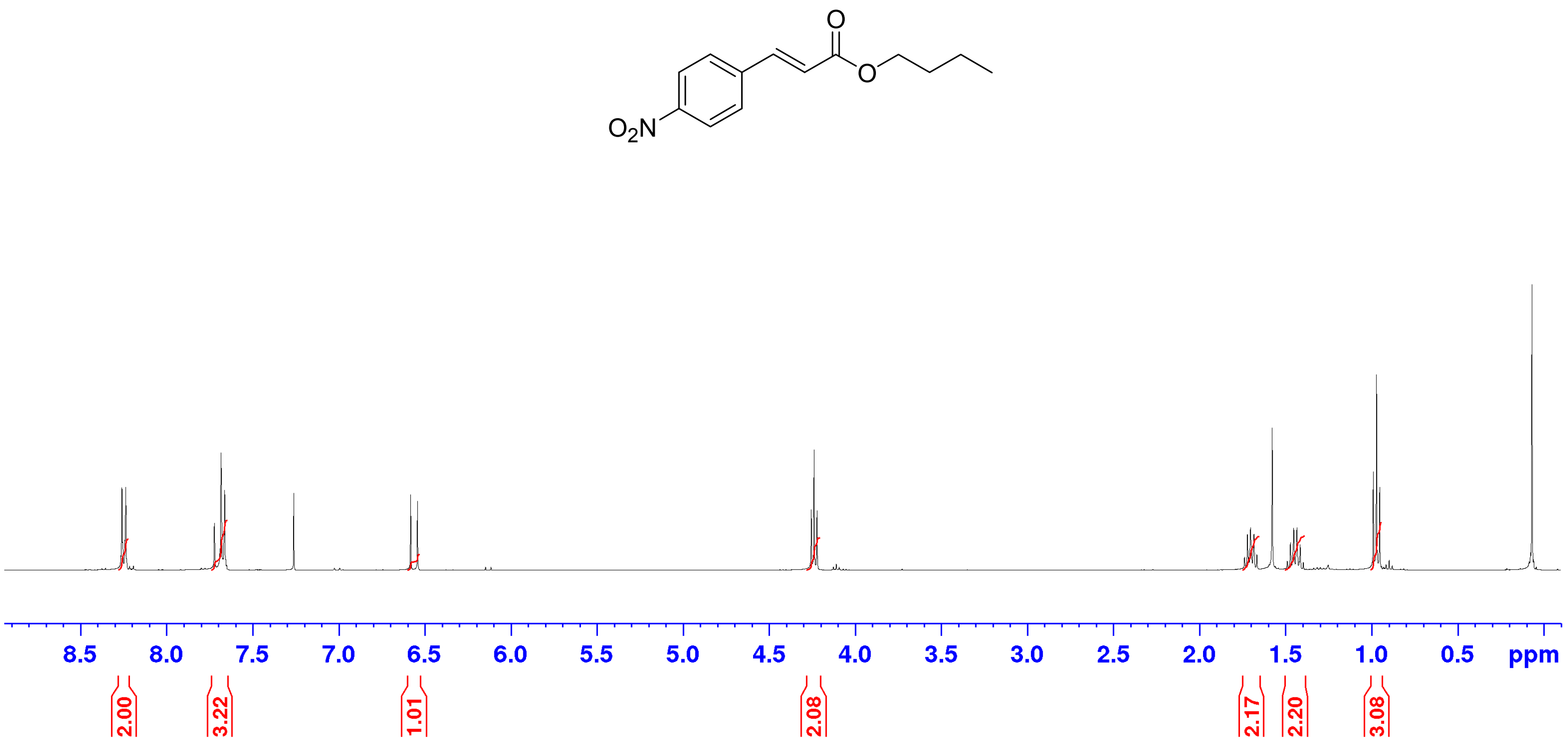

Figure S2. ^{1}H-NMR of 3-(4-nitrophenyl)acrylic acid *n*-butyl ester.

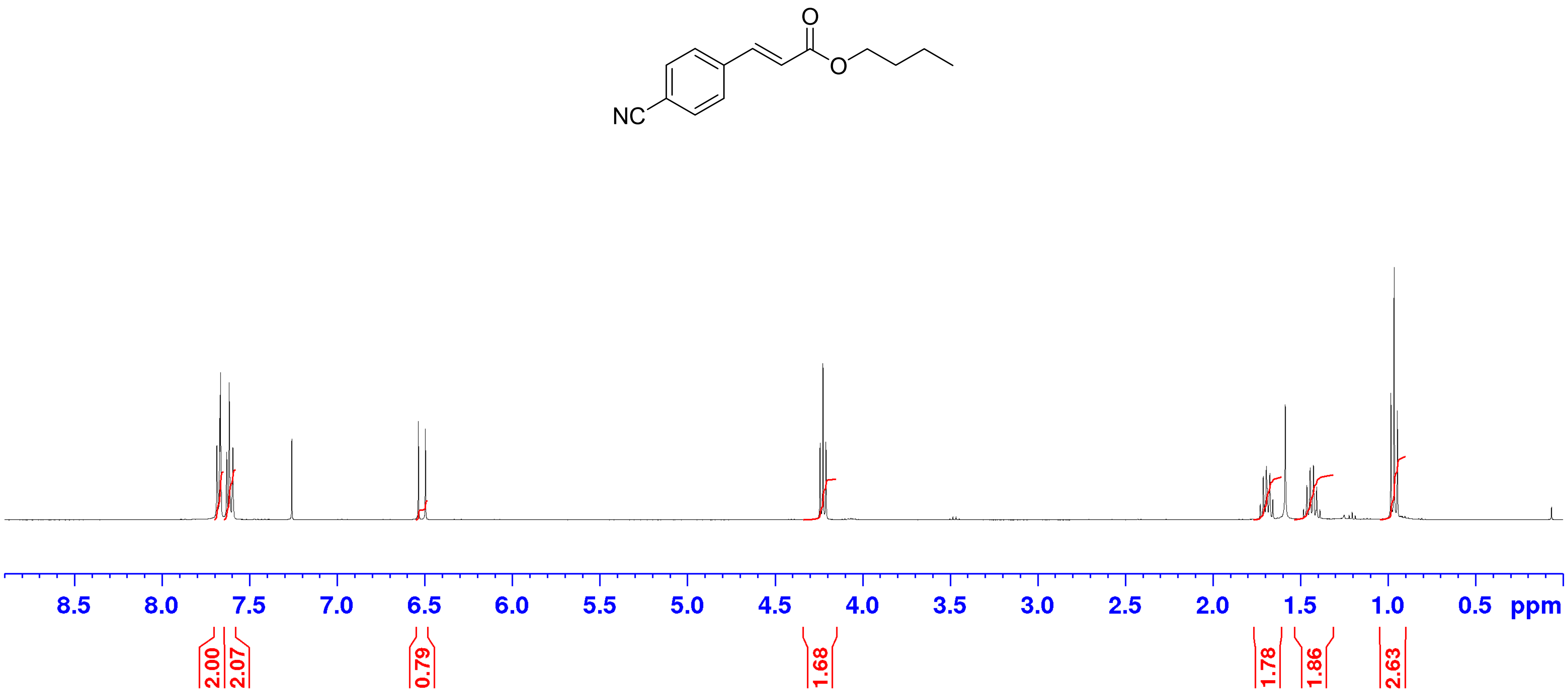

Figure S3. ^{1}H-NMR of 3-(4-cyanophenyl)acrylic acid *n*-butyl ester.

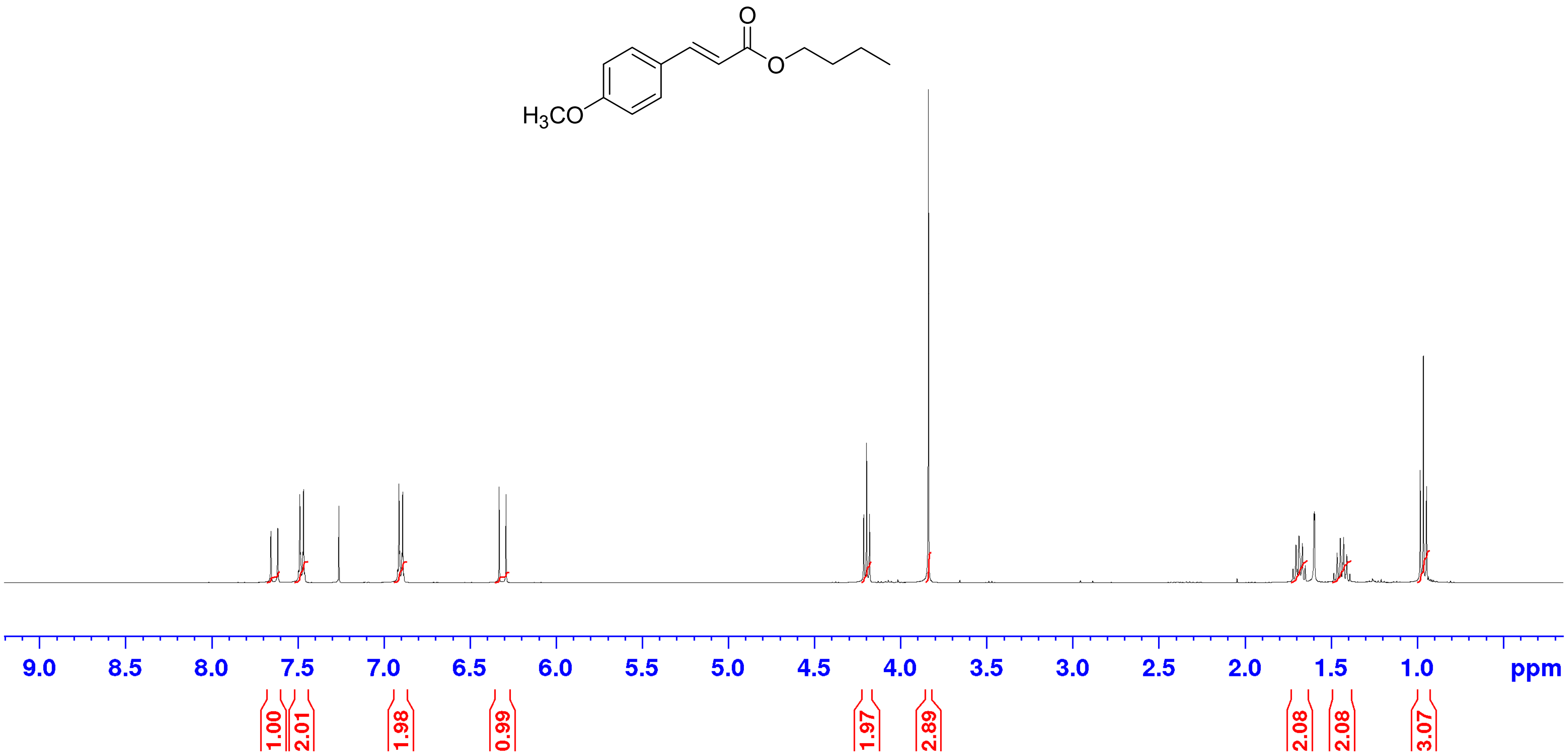

Figure S4. ^{1}H-NMR of 3-(4-methoxyphenyl)acrylic acid *n*-butyl ester.

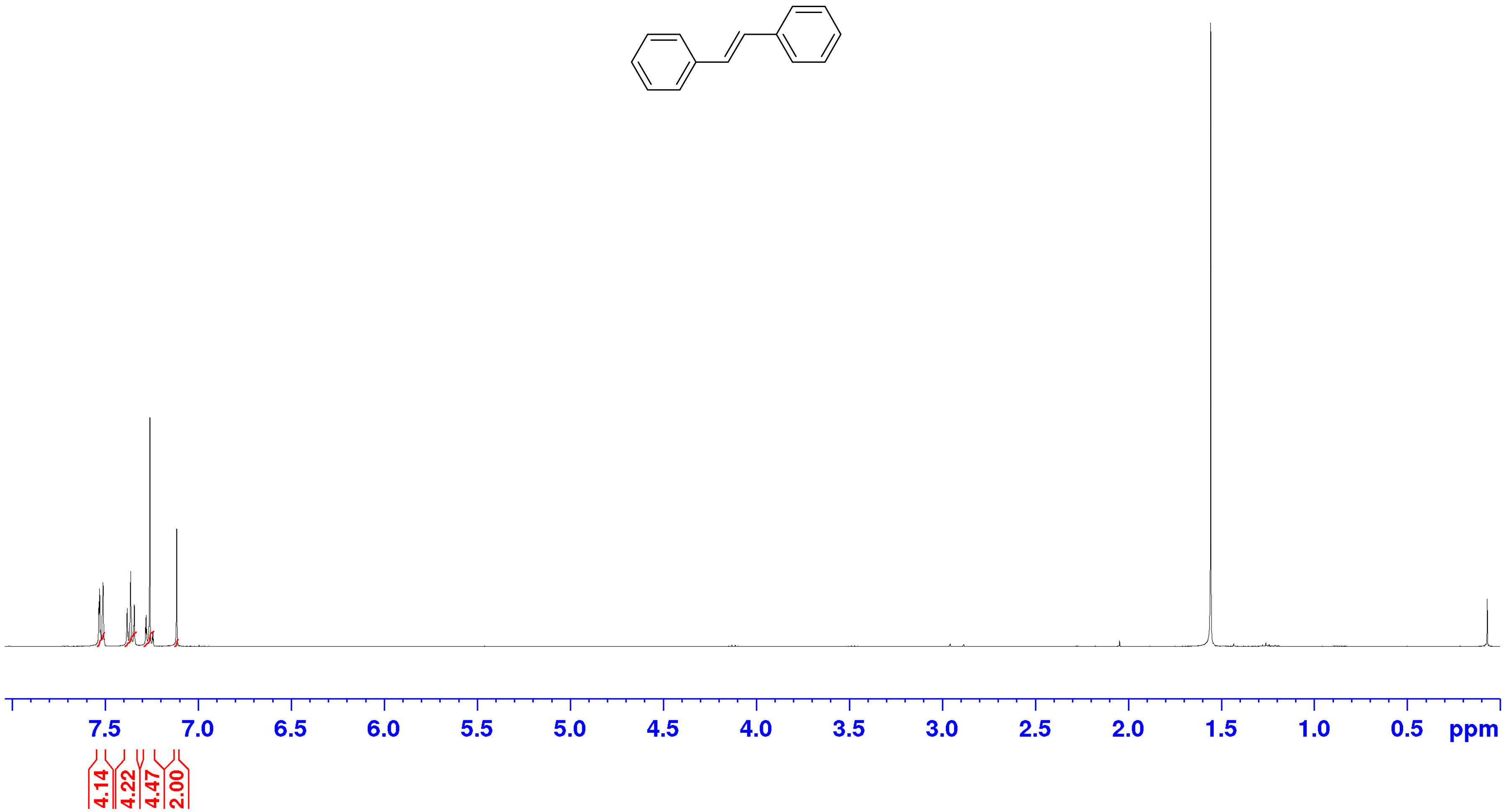

Figure S5. ^{1}H-NMR of stilbene.

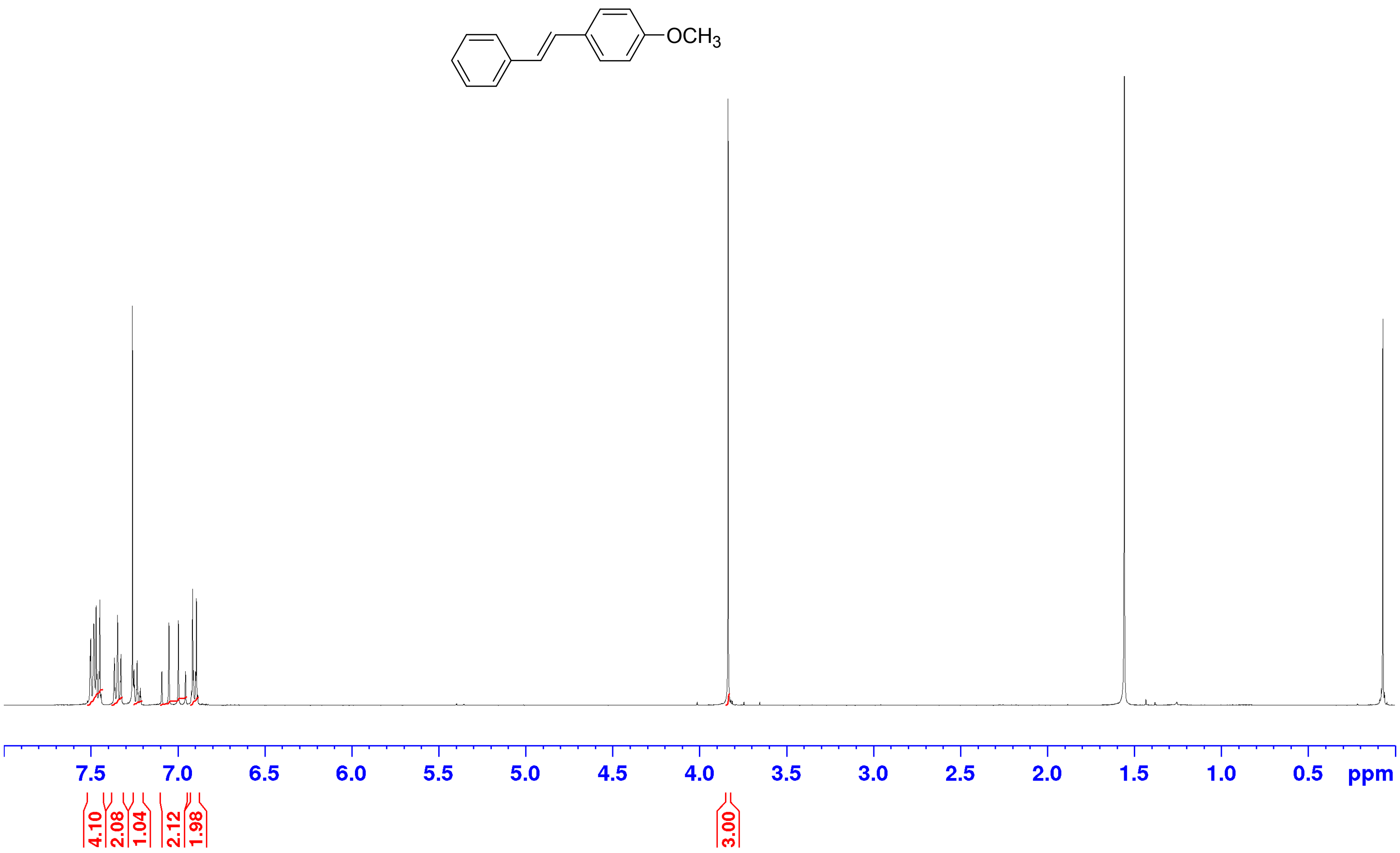

Figure S6. ^{1}H-NMR of 4-methoxystilbene.

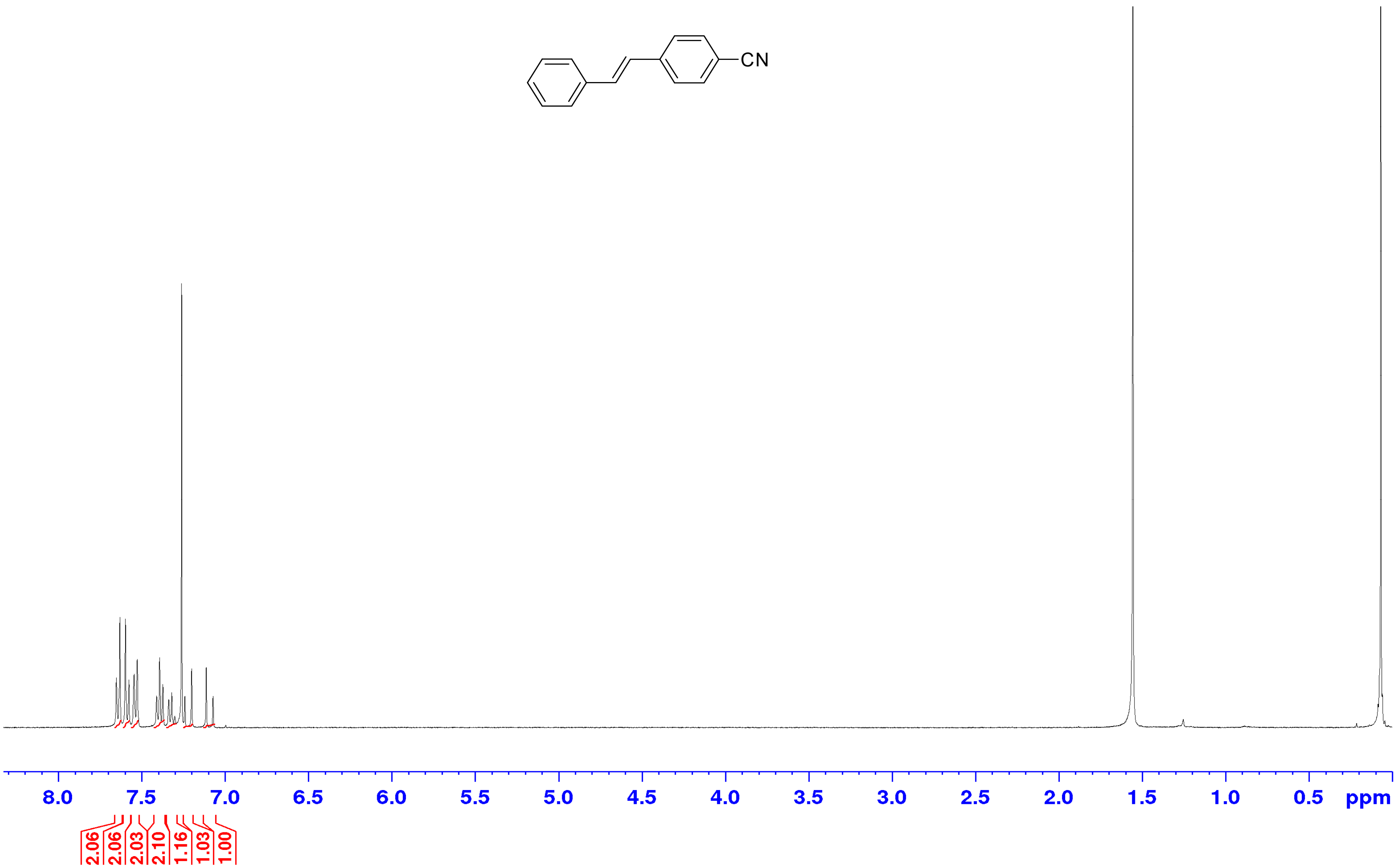

Figure S7. ^{1}H-NMR of 4-cyanostilbene.

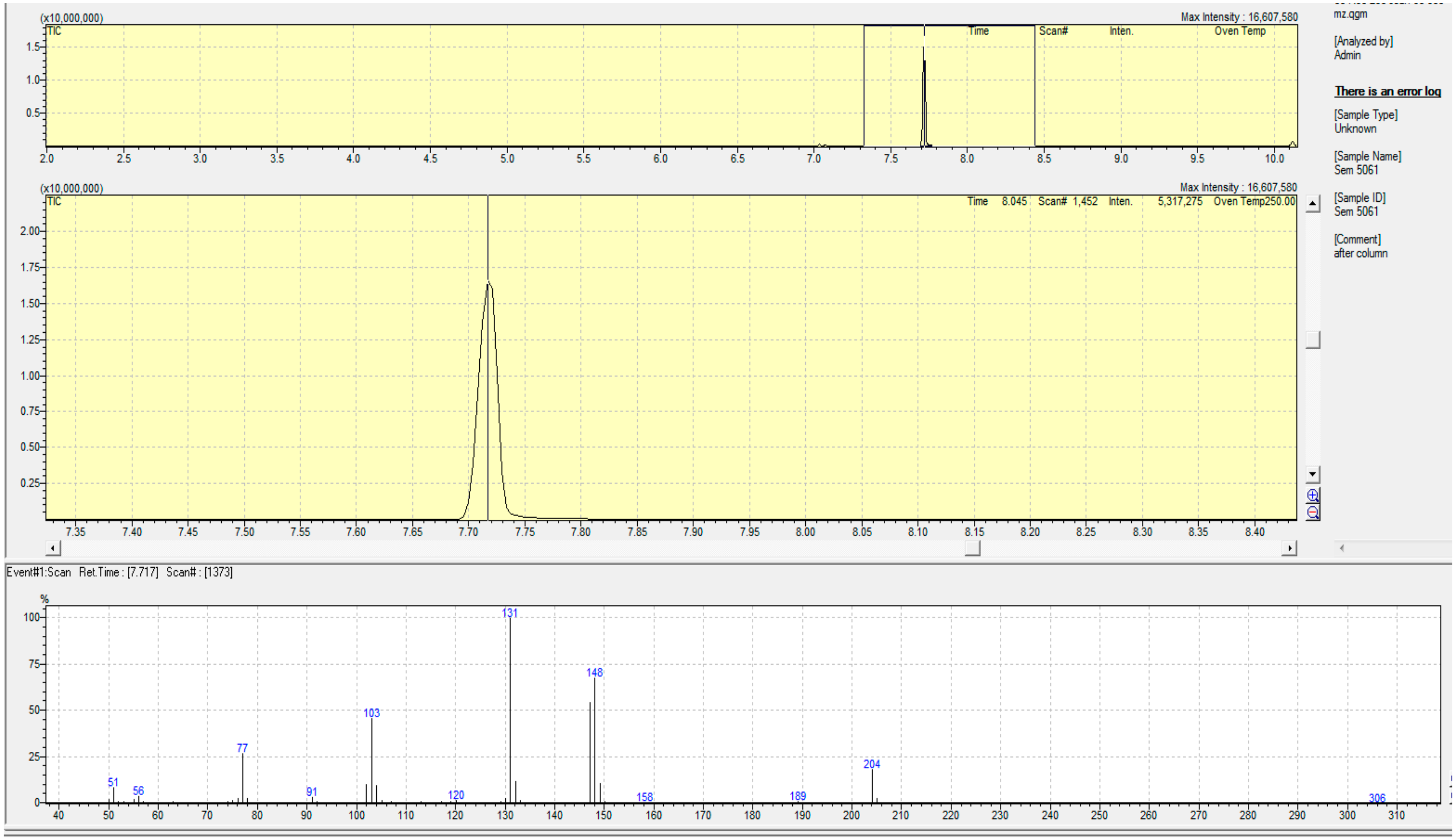

Figure S1. GC-MS data of 3-phenylacrylic acid *n*-butyl ester. m/z = 204.

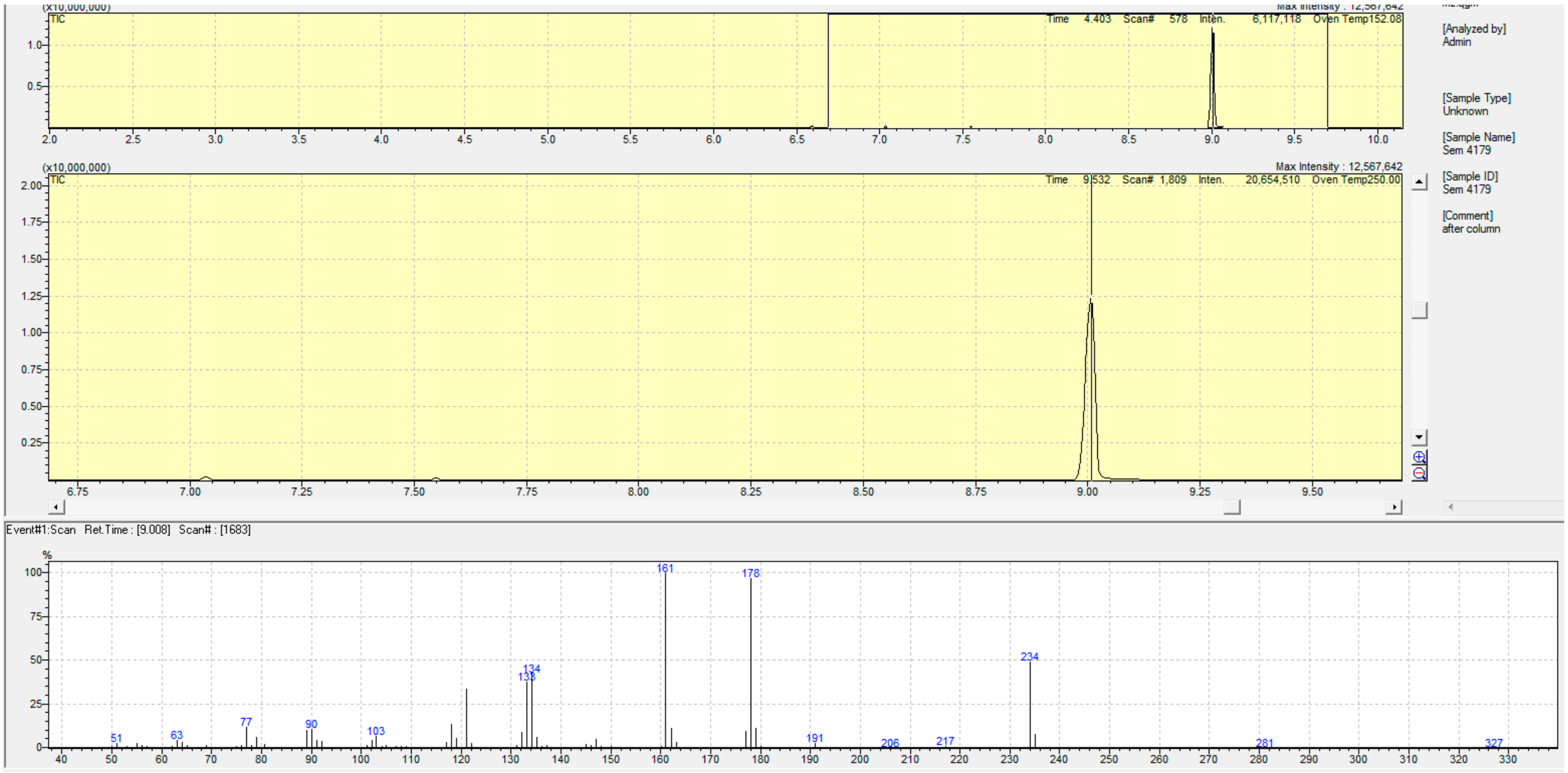

Figure S9. GC-MS data of 3-(4-methoxyphenyl)acrylic acid *n*-butyl ester. m/z = 234.

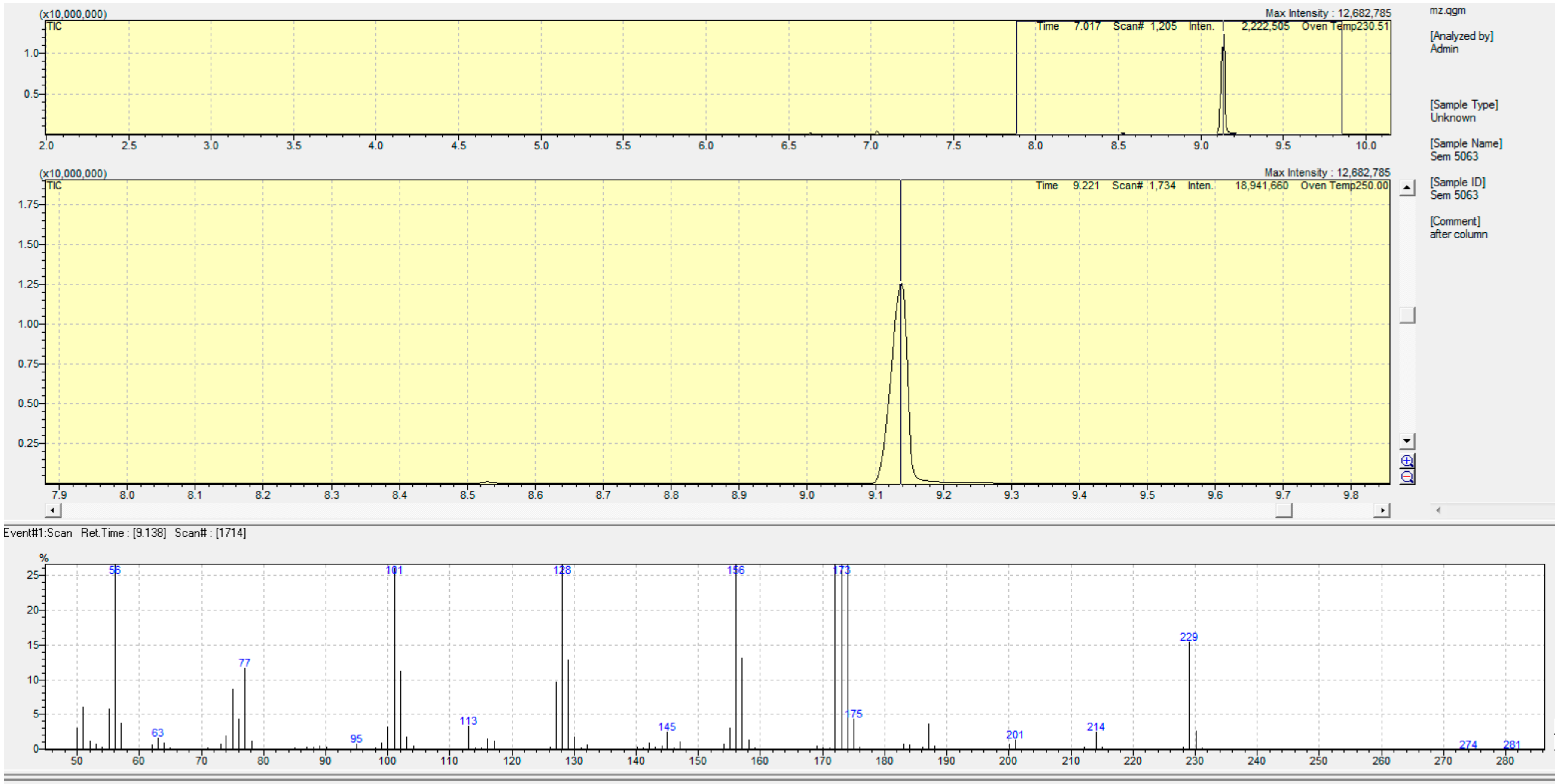

Figure S10. GC-MS data of 3-(4-cyanophenyl)acrylic acid *n*-butyl ester. m/z = 229.

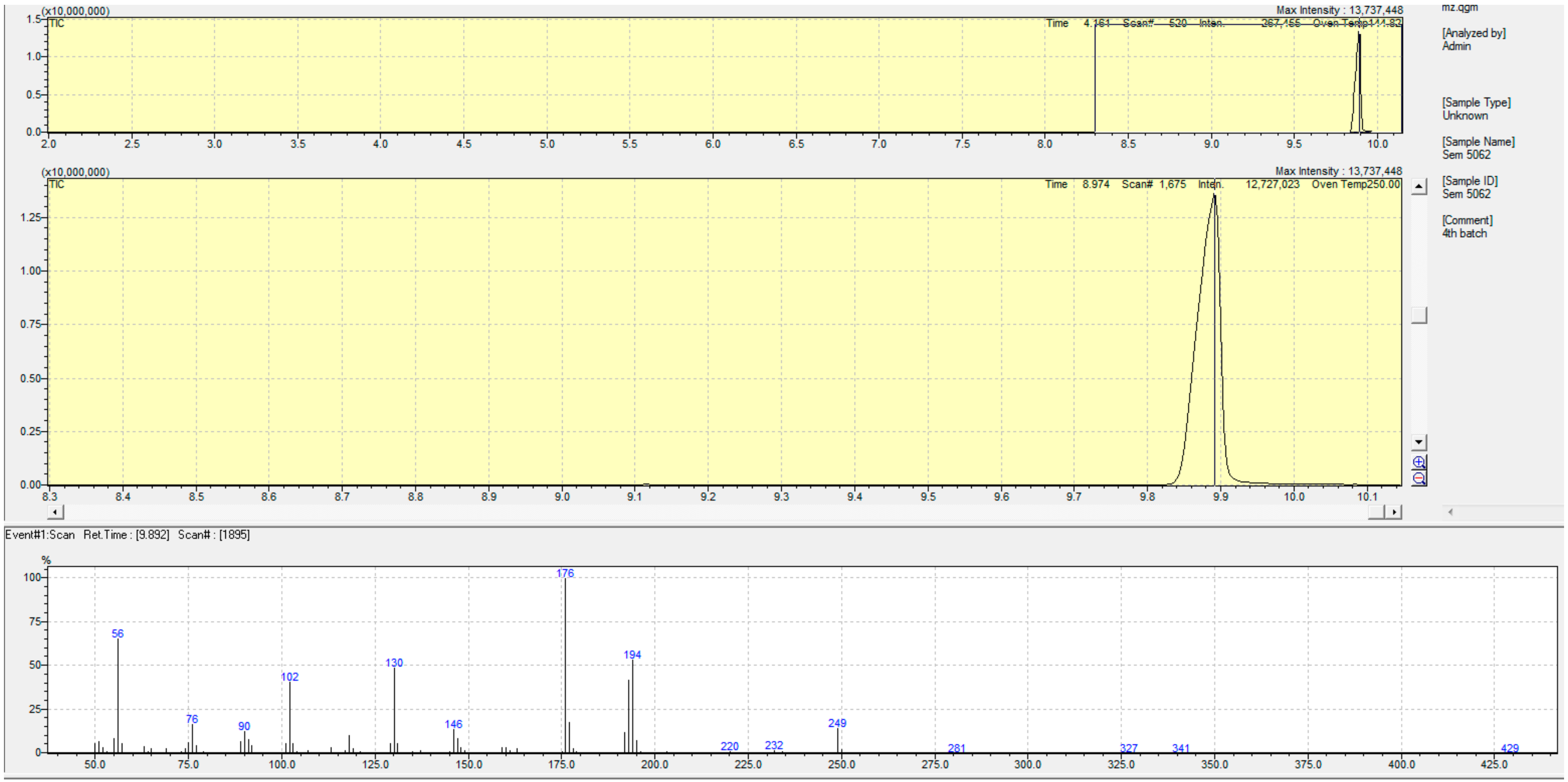

Figure S2. GC-MS data of 3-(4-nitrophenyl)acrylic acid *n*-butyl ester. $m/z = 249$.

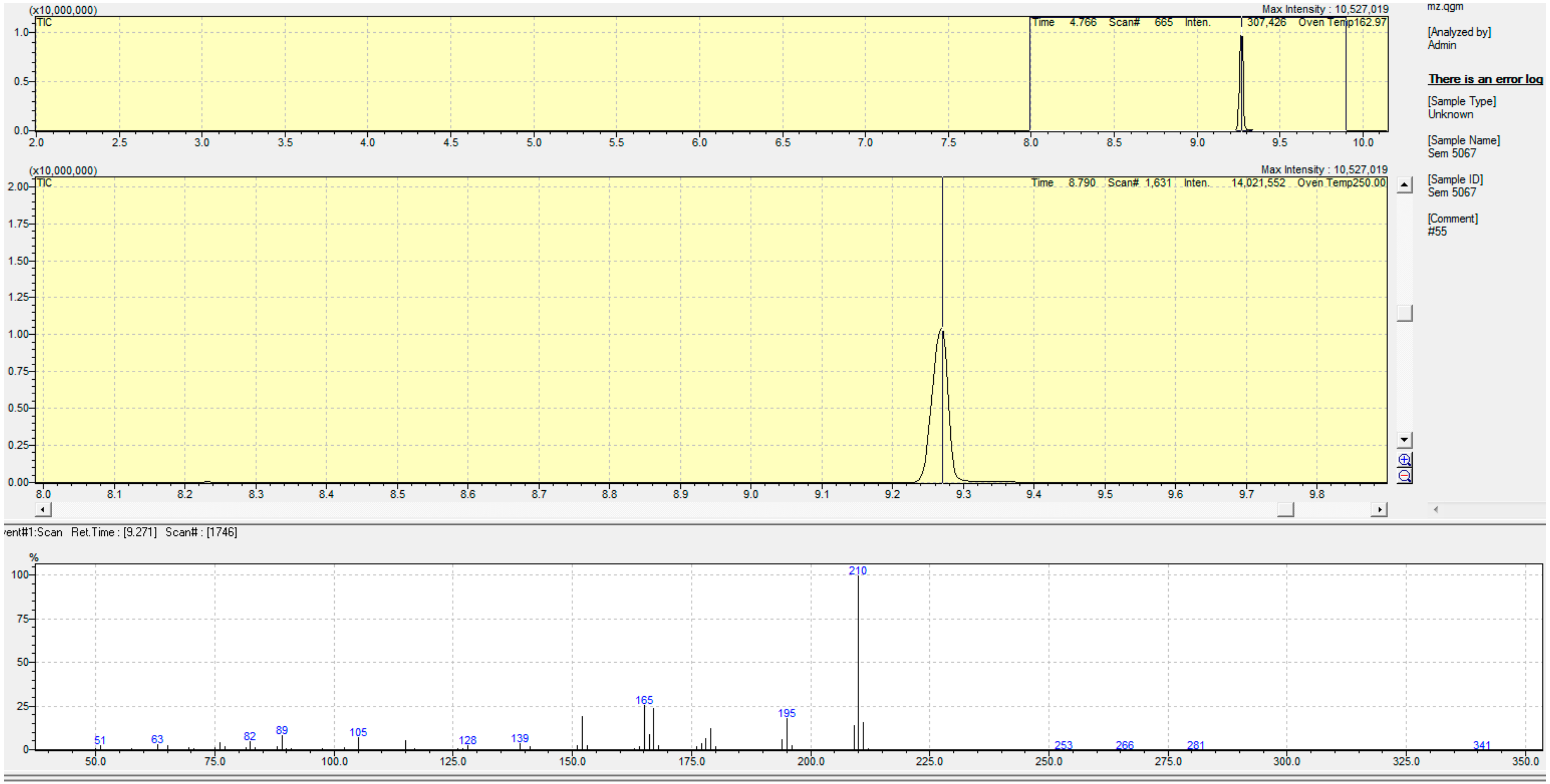

Figure S12. GC-MS data of 4-methoxystilbene. *m/z* = 210.

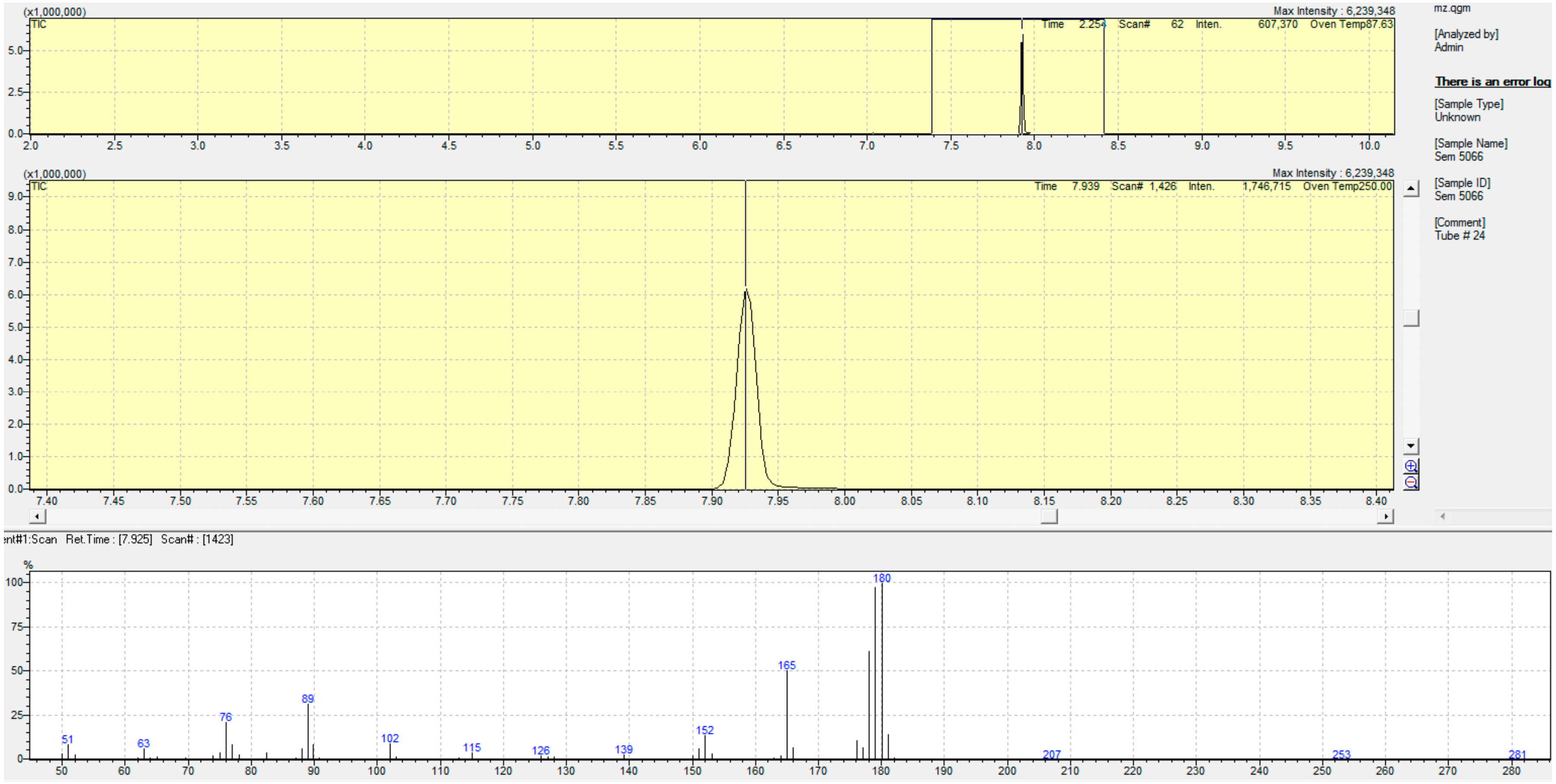

Figure S13. GC-MS data of stilbene. *m*/*z* = 180.

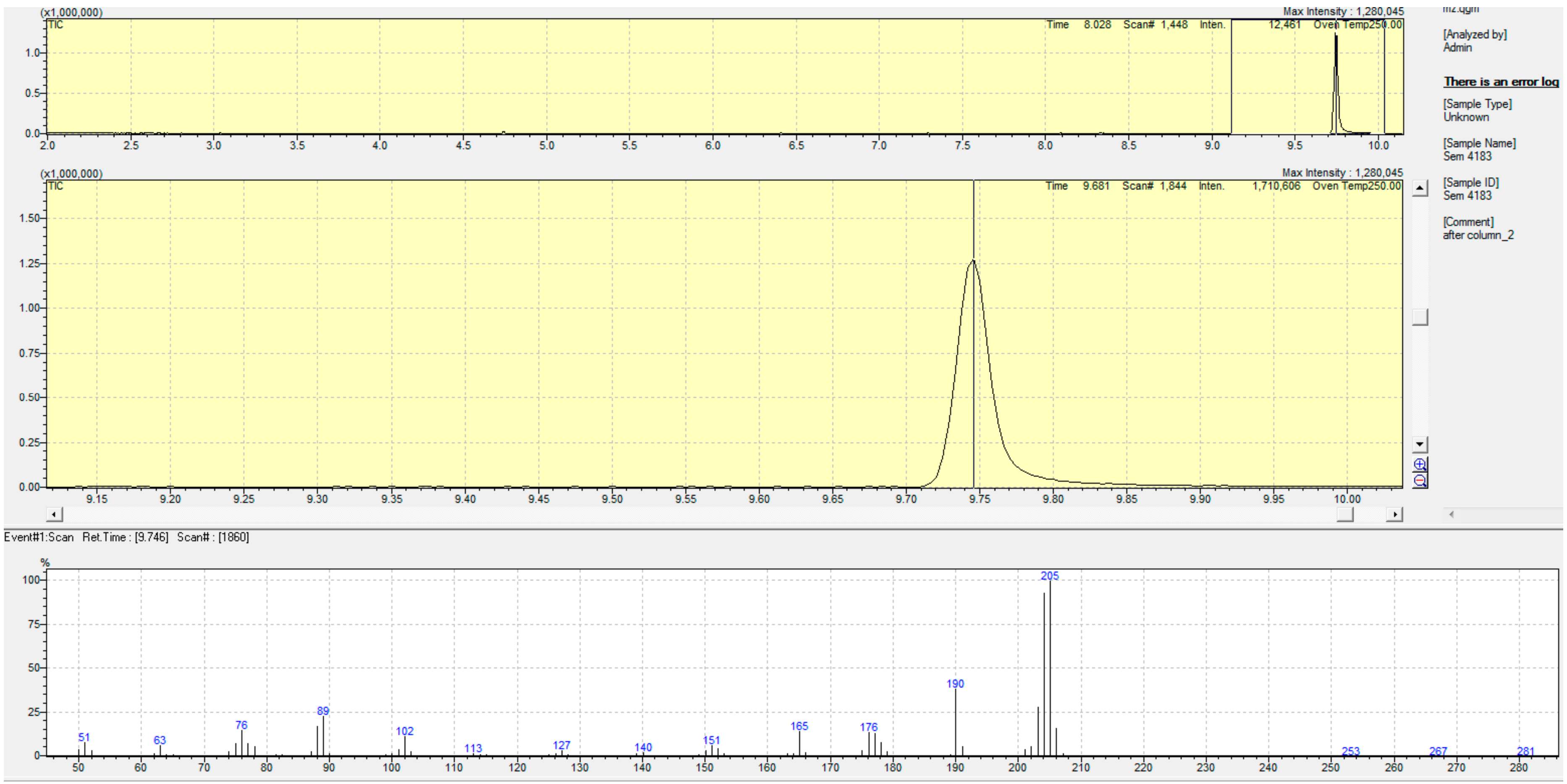

Figure S14. GC-MS data of 4-cyanostilbene. $m/z = 205$.
